# Use of Plasma-Synthesized Nano-Catalysts for CO Hydrogenation in Low-Temperature Fischer–Tropsch Synthesis: Effect of Catalyst Pre-Treatment

**DOI:** 10.3390/nano8100822

**Published:** 2018-10-12

**Authors:** James Aluha, Stéphane Gutierrez, François Gitzhofer, Nicolas Abatzoglou

**Affiliations:** Department of Chemical and Biotechnological Engineering, Université de Sherbrooke, Sherbrooke (Québec), J1K 2R1 Canada; james.aluha@usherbrooke.ca (J.A.); stephane.gutierrez@usherbrooke.ca (S.G.); francois.gitzhofer@usherbrooke.ca (F.G.)

**Keywords:** nano-catalysts, plasma synthesis, pre-treatment, CO-hydrogenation, low-temperature Fischer–Tropsch

## Abstract

A study was done on the effect of temperature and catalyst pre-treatment on CO hydrogenation over plasma-synthesized catalysts during the Fischer–Tropsch synthesis (FTS). Nanometric Co/C, Fe/C, and 50%Co-50%Fe/C catalysts with BET specific surface area of ~80 m^2^ g^–1^ were tested at a 2 MPa pressure and a gas hourly space velocity (GHSV) of 2000 cm^3^ h^−1^ g^−1^ of a catalyst (at STP) in hydrogen-rich FTS feed gas (H_2_:CO = 2.2). After pre-treatment in both H_2_ and CO, transmission electron microscopy (TEM) showed that the used catalysts shifted from a mono-modal particle-size distribution (mean ~11 nm) to a multi-modal distribution with a substantial increase in the smaller nanoparticles (~5 nm), which was statistically significant. Further characterization was conducted by scanning electron microscopy (SEM with EDX elemental mapping), X-ray diffraction (XRD) and X-ray photoelectron spectroscopy (XPS). The average CO conversion at 500 K was 18% (Co/C), 17% (Fe/C), and 16% (Co-Fe/C); 46%, 37%, and 57% at 520 K; and 85%, 86% and 71% at 540 K respectively. The selectivity of Co/C for C_5+_ was ~98% with 8% gasoline, 61%, diesel and 28% wax (fractions) at 500 K; 22% gasoline, 50% diesel, and 19% wax at 520 K; and 24% gasoline, 34% diesel, and 11% wax at 540 K, besides CO_2_ and CH_4_ as by-products. Fe-containing catalysts manifested similar trends, with a poor conformity to the Anderson–Schulz–Flory (ASF) product distribution.

## 1. Introduction

Carbon is a very fascinating element because in the recent past, there has been substantial evidence showing how the final carbon nanomaterial, its growth process, structural morphology and microstructure can be modified by experimental parameters such as the source of carbon feedstock, gas flow rate, synthesis temperature, and the type of catalyst used, including its composition, shape, and particle size [1]. Due to the exceptional chemical, mechanical, electrical, and thermal properties of carbon, its derivative nanostructures have been utilized in diverse fields [2], including the development of semiconductors and application in electronics [3], production of nano-composite materials [4], and chemically active sensors [5]. Magnetic carbon nanotubes (CNTs) can be found in biomedical applications [6], carbon nanotropes for drug delivery [7], CNTs in field emission devices [8], super-capacitors and batteries for energy storage [9], and high-performance energy conversion in solar cells and fuel cells [10]. In all these emerging fields, the properties of novel nanometric materials exhibit substantial variation from the bulk solid state due to their diminished size, for example, in data storage devices and sensors, finely divided magnetic nanoparticles are most desirable [11].

Today, carbon-supported catalysts are receiving attention, especially in regard to their application in the production of synthetic automobile fuels through the Fischer–Tropsch synthesis (FTS) process [12]. Some authors have indicated that the presence of graphitic carbon in such catalysts seems to enhance the hydrocarbon chain-growth probability [13]. The methods used to prepare these FTS catalysts vary greatly, from incipient wetness impregnation [14] to micro-emulsion [15], sol-gel [16], or colloidal synthesis, coupled with the chemical reduction of the metal salts [17]. Sometimes, a combination of known methods is employed, which may involve precipitation and/or impregnation steps [18], or co-precipitation of the metal salts [19]. Other catalyst preparation methods include electrospinning [20], ion-exchange [21], pulsed electron beam ablation (PEBA) [22], carbon-vapor deposition (CVD) [23], and the spray-drying technology [24]. In other works, the single roller melt-spinning method has been used, which involves in situ carbidation through rapid quenching of skeletal nano-crystalline Fe [25]. 

The production of FTS catalysts by induction suspension plasma-spray (SPS) technologies have indicated the potential for commercialization [26]. Plasma technologies are becoming attractive due to the shortened catalyst preparation time involved, in addition to the lower energy requirements, production of highly distributed active species, enhanced selectivity, and catalyst lifetime [27]. Other characteristics presented by plasma synthesis include superior catalyst performance, with the activity and selectivity of the catalysts being higher than those for catalysts prepared by impregnation technology [28]. One disadvantage of the wet chemistry techniques that the plasma technologies can easily overcome, is the requirement for stringent control of numerous synthesis parameters and conditions [29], which lowers the process efficiency. This is far and above the need for synthesizing the catalyst support material separately from the active phase that finally demands multi-step activation [30]. 

Efforts to synthesize FTS catalysts through plasma commenced in the 1980s [31], enabling both single-metal and bimetallic Co-Fe formulations to be produced [32]. Advanced materials such as the photocatalytic Au-Ag core-shell nanoparticles have been synthesized using plasma, whose application today goes beyond the production of FTS catalysts [33]. In fact, plasma techniques can successfully be used both during the catalyst synthesis period [34], and at the pre-treatment stage to activate the catalyst [35]. For example, as used in conventional calcination instead of applying excessively high pre-treatment temperatures such as 973–1473 K [36], the as used in conventional calcination, plasma-glow discharge (PGD) could be applied to lower the pre-treatment temperatures and still produce smaller Co metal nanoparticles (<7 nm) [37], with a significant improvement to metal dispersion as the particle size is shown to be a function of the PGD intensity [38]. 

Similarly, the dielectric-barrier discharge (DBD) plasma has been seen to promote FTS over Cu/Co-based catalyst, at ambient pressure and much lower temperatures because they strongly suppress CH_4_ production [39]. Non-thermal plasma (NTP) reactors, on the other hand, are currently being considered as a viable alternative to the conventional FTS process because they perform reactions rapidly, and may operate at ambient temperature, with or without a catalyst, in minimal space, and at a low cost of maintenance [40]. This is because NTP reactors generate new reactive species through plasma-photon emissions or thermal hot-spots that can initiate catalytic reactions when plasma is combined with the catalyst [41]. 

Since plasma techniques present many positive effects leading to improved structural properties of FTS catalysts, better metal dispersion, smaller metal cluster size, and more uniform particle size distribution, they also decrease operational temperatures in FTS, with higher CO hydrogenation activity, and better suppression of CH_4_ formation and coke deposition [42]. Therefore, the current advancement in the production of synthetic fuels through FTS involves the application of induction SPS technology in producing nanometric C-supported catalysts that inherently consist of active catalytic species for FTS using Fe-based catalysts [26], as well as Co-based, and modified Co-Fe catalysts [43]. 

This paper is written in the context of earlier work and the novelty of this study encompasses the production of C-supported multi-component FTS catalysts, which further generate structural variations in the catalysts when different pre-treatment procedures are employed. In this investigation, a comparative study was conducted for FTS activity using three different plasma-synthesized catalysts supported on carbon (that is, Co/C, Fe/C, and 50%Co-50%Fe/C formulations). The materials were used to test the effect of (i) temperature, and (ii) the pre-treatment procedure on the FTS product spectrum. An attempt to determine the catalysts’ α-values and the H_2_ utilization efficiency in the process was made. Since some authors have shown that the selectivity of classical FTS catalysts towards CH_4_ is significantly lowered when using a low molar H_2_:CO ratio in the gas feed [44], in this study, changing the pre-treatment (or reduction) procedure of plasma-synthesized catalysts has achieved similar results with H_2_-rich feeds, which is generally unusual. 

## 2. Materials and Methods 

### 2.1. Materials and Catalyst Synthesis

#### 2.1.1. Materials 

The raw materials used in this research included 99.8% Co metal (particle size: 1–10 μm), from Aldrich (Milwaukee, WI, USA ); 99.9+% Fe metal (1–10 μm) from Alfa Aesar (Tewksbury, MA USA); the following high purity gases: H_2_ (N5.0), CO (N2.5), and Ar (N5.0), from PRAXAIR (Sherbrooke, QC, Canada); mineral oil with catalog name “O122-4, Mineral Oil, Heavy; USP/FCC (Paraffin Oil, Heavy)” from Fisher Scientific (Ottawa, ON, Canada), and 99% squalane solvent from Sigma-Aldrich, (Oakville, ON, Canada).

#### 2.1.2. Catalyst Synthesis by Plasma 

The plasma reactor used in catalyst synthesis is a high frequency (HF) 60 kW SPS system operating on a plasma torch supplied by Tekna Inc. (Sherbrooke, QC, Canada), with the PL-50 coil and the subsonic nozzle. In the sheath of the flame, the gas flow rates were set at 75 SLPM for Ar and 10 SLPM for H_2_ while the other Ar gas flow rates were 23 SLPM (Central gas) and 10.4 SLPM (Powder). The voltage was set at ~6.6 kV, current 4.4 A, and 0.5 A (grounding) to provide an approximate 29-kW power output.

In catalyst preparation, a mass of 60 g of the metal (Co-only, Fe-only or both in a predetermined ratio, such as 50-50) were mixed with 300 cm^3^ of mineral oil by stirring for at least two hours in order to form a homogeneous suspension. This suspension was then injected directly into the plasma at a flow rate of 8.2 cm^3^.min^−1^. The plasma equipment is designed in such a way that samples may be collected from both the primary (main) plasma reactor and the secondary (auxiliary) vessel serving to quench the exiting gas [43]. Samples from each of these vessels were collected separately, although they are generally identical in nature by chemical composition. However, certain subtle differences such as particle size may cause their separation into the different vessels. In this study, samples of equal mass were drawn from each vessel and mixed homogeneously before being tested.

### 2.2. Catalyst Testing and Experimental Methods

#### 2.2.1. Catalyst Activity Testing 

The catalysts were tested for Fischer–Tropsch activity in a 0.5 L purpose-made 3-phase-continuously stirred tank slurry reactor (3-φ-CSTSR) supplied by Autoclave Engineers (Erie, PA, USA). The initial results for single metal Co/C and Fe/C catalysts were benchmarked against the commercial Fe/C catalyst and the results can be found in our earlier publication [45]. In addition, details of the testing methodology and instrumentation for the data acquisition defining catalyst activity by CO conversion in FTS can be found in the same article. 

In this work, the plasma-synthesized catalysts (7.5 g each) were activated using several reducing media in succession. The pre-treatment was necessary in order to gasify the excess carbon matrix from the metal moieties. The materials were reduced at a constant temperature of 673 K (400 °C) in H_2_ flowing at 250 cm^3^.min^–1^ for 24 h, followed by CO for 10 h and then H_2_ again for 10 h. After cooling and purging with inert (Ar), the liquid phase was introduced into the vessel using 250 cm^3^ of squalene solvent before CO hydrogenation was then conducted at various temperatures (500, 520, and 540 K) under 2 MPa of pressure. The gas molar ratio used in FTS was H_2_:CO = 2.2 with the gas composition comprising 65% H_2_ and 29% CO balanced in Ar, all flowing at 250 cm^3^.min^–1^ (STP), giving a gas hourly specific velocity of GHSV = 2000 cm^3^.h^–1^.g^–1^ of the catalyst.

#### 2.2.2. Catalyst Selectivity Determination

Two dedicated offline Varian CP-3800 Gas Chromatographs from Varian, Inc., (Walnut Creek, CA, USA), were used to determine the catalysts’ selectivity. By integrating the area under each product peak, one GC was used to calculate the selectivity of the gaseous products (e.g., CO_2_, CH_4_, C_2_H_6_). After a period of 24 h of catalyst testing, the slurry was sampled, filtered, and injected into the second GC, with selectivity towards each hydrocarbon being determined by peak integration. Full details of the method and approach used to establish the catalyst selectivity can be found in an earlier publication [46]. From the results obtained, plots of selectivity against the hydrocarbon chain length were used to determine the α–values of the catalysts, calculated from their kinetic data using Equations (1) and (2) of the Anderson–Schulz–Flory (ASF) model as done before [47], but in this study, it is done under different FTS reaction conditions.

(1) $$\;\frac{M_{n}}{n}=\left(1-\alpha \right){}^{2}.\alpha^{\left(n-1\right)}\;$$

(2) $$\;\ln\left(\frac{M_{n}}{n}\right)=n\ln\alpha +\ln\left[\frac{{\left(1-\alpha \right)}^{2}}{\alpha }\right]\;$$

where:

M_n_ = mole fraction of a hydrocarbon with chain length n
n = total number of carbon atoms in the hydrocarbon chain
α = probability of chain growth (α < 1)
(1 − α) = probability of chain termination

### 2.3. Catalyst Characterization

#### 2.3.1. BET Surface Area Analysis 

The fresh samples were characterized by the Brunauer-Emmett-Teller (BET) surface area analysis using an Accelerated Surface Area Porosimeter (ASAP 2020) from Micromeritics Instrument Corp. (Norcross, GA, USA). Full details of the analysis procedure and test conditions for the BET specific surface area determinations are available in an earlier publication [43]. However, in summary, the samples were degassed at 383 K (110 °C) for 16 h until a pressure of less than 10 μm Hg (1 Pa) was obtained in the sample holder, and BET physisorption was carried out using N_2_ gas under liquid N_2_ at 77 K (−196 °C).

#### 2.3.2. Microscopic Analysis 

The Hitachi S-4700 Scanning electron microscope (SEM) from Hitachi High-Technologies Corp. (Tokyo, Japan) was used to examine the morphological properties of the catalysts, capturing both secondary and backscattered images. An inbuilt X-Max Oxford EDX (energy dispersive X-ray) spectrometer coupled to the SEM (Hitachi, Tokyo, Japan) was used for elemental analysis, while X-ray elemental mapping visually indicated the degree of dispersion of the metals in the carbon matrix. Transmission electron microscopy (TEM) was conducted on a Hitachi H-7500 instrument supplied by Hitachi High-Technologies Corp. (Tokyo, Japan), with sample images captured by means of a bottom-mounted AMT 4k x 4k CCD Camera System Model X41, and the analysis details are available in earlier works [48]. The Nano-measurer version 1.2 “Scion Imager” software was used to analyze the metal nanoparticle size distribution. In order to determine whether a significant difference existed between the various population sets in the particle size analysis, a *t*-test was applied using grouped data of 300 metal nanoparticles each.

#### 2.3.3. X-ray Photoelectron Spectroscopy (XPS) 

Elemental composition and the oxidation states of the fresh catalysts were determined by an XPS Kratos Axis Ultra DLD spectrometer from PANalytical B.V. (Almelo, The Netherlands), with sample excitation coming from the monochromatized Al*Kα* line (1486.6 eV) with applied power of 225 W. The analyzer operated in a constant pass energy mode with *PE* = 160 eV for the survey scans and *E*pass = 20 eV for the high-resolution scans. The work function of the instrument was calibrated to give a binding energy (BE) of 83.96 eV for the 4f7/2 line of metallic Au. The dispersion of the spectrometer was adjusted to a BE of 93.62 eV for the 2p3/2 line of metallic Cu. The powdered catalysts were mounted on non-conductive adhesive tape. A charge neutralizer was used on all samples to compensate for the charging effect. Charge corrections were done using the graphitic peak set at 284.5 eV. 

The Casa XPS software (version 2.3.18) was employed for data analysis. The fitting parameters for the high-resolution Fe 2p and Co 2p spectra were derived from the literature [49]. Since the XPS machine used in the present analysis was similar to the model used in the reference under identical experimental conditions [50], the asymmetric model specified by the XPS reference webpages was used for the fitting parameters of the graphitic C 1s. Data fitting was performed to ensure that the models were within a standard deviation of less than 2, as indicated in our earlier work [48]. 

#### 2.3.4. X-ray Diffraction (XRD) Analysis 

The Philips X’pert PRO Diffractometer from PANalytical B.V. (Almelo, The Netherlands) was used for the powder-XRD analysis in this study. Having been fitted with Ni-filters for the Cu Kα radiation produced at 40 kV and 50 mA with wavelength alpha1 as (λ = 1.540598 Å), the instrument was set in the Bragg-Brentano configuration with a proportional Xe point detector, and the diffractometer was operated on the factory-installed Analytical Data Collector software. The XRD patterns were recorded in the range of 5° and 110° [2θ] for an acquisition time of 4 h per sample. Data collection and analysis was conducted using the Materials Data Inc. software: the MDI JADE 2010 (version 6.7.0 @ 2018-01-31), and the collected data were compared with the Powder Diffraction Files in the Database (version 4.1801) using the PDF-4+ software 2018 (version 4.18.02), published by the International Centre for Diffraction Data (ICDD). A Rietveld quantitative analysis (RQA), which involves quantification of each phase in the material was modeled using the HighScore Plus software (V4.7) in conjunction with the XRD analysis, and details of the methodology used are provided in our earlier works [46]. Characterized by Equation (3), the curve fitting for RQA attempted to determine the amount of each species in the used catalysts [51].

(3) $$\;W_{p}=\frac{S_{p}{\left(\mathrm{ZMV}\right)}_{p}}{\sum_{i=1}^{n}S_{i}{\left(\mathrm{ZMV}\right)}_{i}}\;$$

where

W_p_ = relative weight fraction of phase p in a mixture of n phases,
S_p_ = Rietveld scale factor,
Z = number of formula units per cell,
M = mass of the formula unit (in atomic mass units), and
V = the unit cell volume (in Å^3^).

## 3. Results

### 3.1. Catalyst Testing

#### 3.1.1. Activity Determination by CO and H_2_ Conversion 

**Figure 1:**
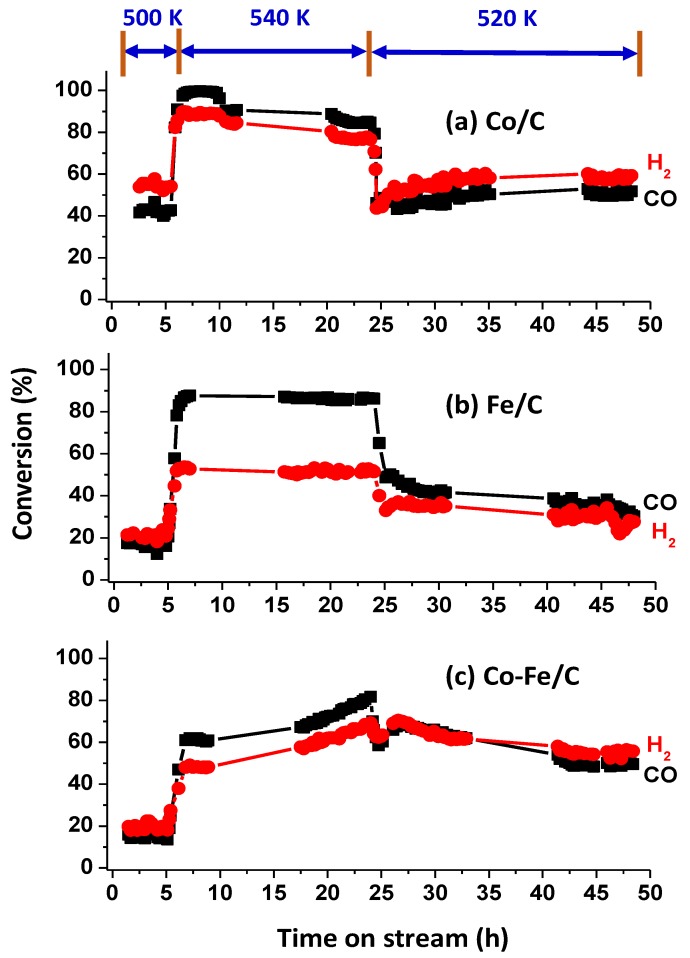
The activity of (**a**) Co/C, (**b**) Fe/C, and (**c**) Co-Fe/C catalysts tested at 500 K, 520 K, and 540 K (pressure = 2 MPa; GHSV = 2000 cm^3^ h^−1^ g^−1^ of the catalyst) indicating the CO and H_2_ conversions.

Figure 1 shows that a rise in temperature increased catalyst activity for CO hydrogenation, with FTS operating at 2 MPa pressure and GHSV = 2000 cm^3^.h^−1^.g^−1^ of the catalyst. An identical temperature profile was used in catalyst testing at 500 K for the first 5 h, then ramped and held at 540 K for the next 19 h, and finally dropped to 520 K and held there for another 24 h. 

The respective CO conversions recorded (at 500 K, 520 K, and 540 K) averaged 18%, 46%, and 85% for the Co/C catalyst; 17%, 37%, and 86% for the Fe/C catalyst; and 16%, 57% and 71% for the Co-Fe/C catalyst. It was expected that in a hydrogen-deficient feed stream, high selectivity towards the production of alkenes is most likely to occur as shown by Equation (4), while in hydrogen-rich feed streams, as is the case in this study (H_2_:CO = 2.2), selectivity should lean towards the production of alkanes according to Equation (5). It has been observed that CH_4_ production, which is indicated by Equation (6) becomes rampant at elevated temperatures, enhanced by hydrogen-rich feed streams. An active water-gas shift (WGS) catalyst should convert some of the H_2_O generated in Equations (4)–(6) into CO_2_ and H_2_, thereby enriching the H_2_ feed stream as given in Equation (7).

2n H_2_ + n CO → C_n_H_2n_ + n H_2_O (4)

(2n+1) H_2_ + n CO → C_n_H_2n+2_ + n H_2_O (5)

3 H_2_ + CO → CH_4_ + H_2_O (6)

H_2_O + CO → CO_2_ + H_2_ (7)

**Figure 2:**
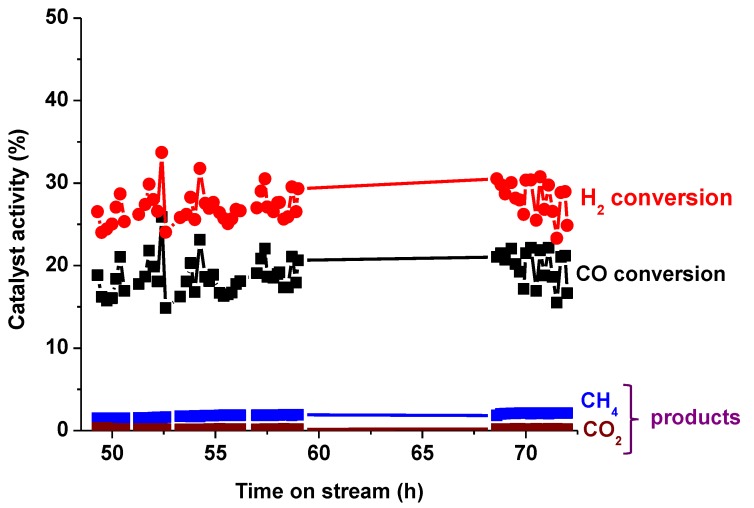
The activity of the Co/C catalyst extended to 72 h on stream at 500 K, 2 MPa and GHSV = 2000 cm^3^ h^−1^ g^−1^ of the catalyst.

**Figure 3:**
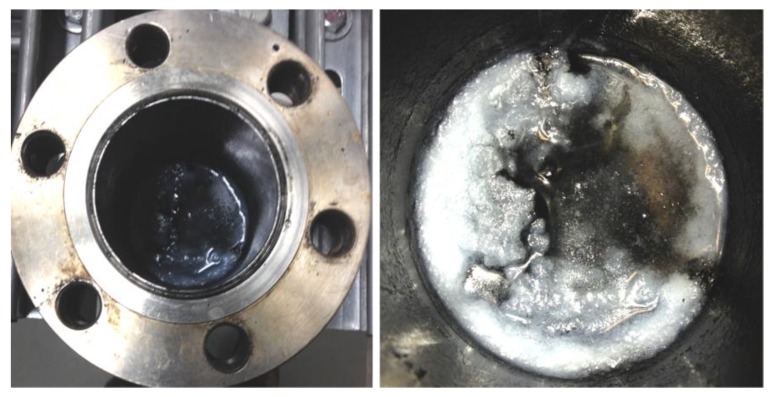
The sample pictures showing a massive wax formation in the reactor after an FTS reaction by carbon-supported catalysts that are evidently black with the wax being conspicuously white.

From the gas-product analysis by GC, generally, there was a significant formation of CH_4_ observed followed immediately by almost a non-existent (C_2_–C_4_) portion, with very little of C_2_H_6_ and C_2_H_4_ observed (amounting to ~1%, when combined), and no C_3_ and C_4_ were detected. The other products from C_5_ and above were analyzed by the liquid-based GC. Figure 2 provides extra data for the Co/C catalyst, which was tested beyond the 48 h window and it shows that the catalytic activity at 500 K stabilized at a much lower value (~18%) than the initial activity recorded within the first 5 h on stream (~40%). It is suspected that the drop in catalytic activity by the third day was probably not a sign of catalyst deactivation, but rather due to the competition for the active sites by both the feed gas and the accumulated FTS products. This is because by the third day the reactor was already full of the FTS products, as shown in Figure 3 by the massive presence of wax in the reactor. Nonetheless, the catalyst showed a remarkably low production of CO_2_ (0.2%) and CH_4_ (1.9%), both of which are considered as undesirable by-products. Although the catalyst may have recorded a lower activity after 48 h of operation probably due to the variation in the reactor environment with time-on-stream (TOS), a lower catalytic activity is usually preferred when accompanied by a high selectivity than with a poor selectivity as the unreacted feedstock in the exit gases can always be recycled. 

Since Figure 1 indicates the perpetual switching of the feed-gas consumption as sometimes the CO conversion was higher, equal, or lower than that of H_2_, dividing the H_2_ conversion by that of CO defined the H_2_:CO uptake ratio. See the summary of results in Table 1., which shows the aggregated fractions as light gases (C_2_–C_4_); gasoline fraction (C_5_–C_12_); diesel (C_13_–C_20_), and waxes (C_21+_).

**Figure 4:**
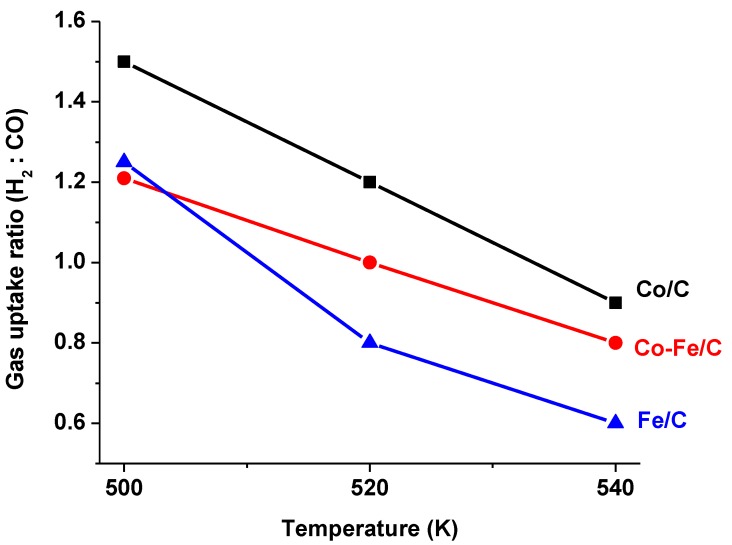
The H_2_:CO ratio, r, which indicates that all the catalysts, at higher temperatures, converted more CO to form CO_2_ and probably with increased water-gas shift, FTS demanded less H_2_.

The evident change in the H_2_ and CO conversions whose ratio waswhich being either below or above equity was probably due to the formation of different products; for example, more H_2_ is required in CH_4_ production, which usually occurs at elevated temperatures, according to Equation (6). Increasing temperature lowered the H_2_:CO uptake ratio gradually as depicted in Figure 4, as follows: 1.5, 1.2, and 0.9 for the Co/C catalyst (at 500 K, 520 K, and 540 K respectively), 1.3, 0.8, and 0.6 for the Fe/C catalyst and 1.2, 1.0, and 0.8 for the 50%Co-50%Fe/C catalyst. This means that Co/C was the most efficient catalyst in the H_2_ utilization and that at lower temperatures, more H_2_ was utilized in CO hydrogenation, while at higher temperatures, a significant quantity of CO converted did not incorporate H_2_ into the FTS products. 

It was evident that at higher temperatures (540 K), more CO_2_ was produced by all the three catalysts (~19% for Fe/C, ~14% for Co/C, and 11% for Co-Fe/C) and presumably with increased water-gas shift, FTS would demand less from the H_2_ feed as the system is enriched with the H_2_, according to Equation (5). One striking observation made was that since the original intention of this study was to test the catalysts for high-temperature (HT)-FTS, which normally operates above 600 K, but the H_2_ utilization was already very poor at 540 K (with large quantities of the CO feed going to CO_2_ and CH_4_). This led to the conclusion that nanometric plasma-synthesized catalysts are well designed for LT-FTS operations only (below 520 K).

#### 3.1.2. Selectivity Results 

As expected, higher temperatures enhanced gasoline production, while lower temperatures enriched the diesel fraction. At 500 K, the Co/C catalyst produced 8% gasoline, 61% diesel, and 28% wax. At 520 K, Co/C produced 22% gasoline, 50% diesel and 19% wax, while at 540 K, it generated 24% gasoline, 34% diesel, and 11% wax. 

**Figure 5:**
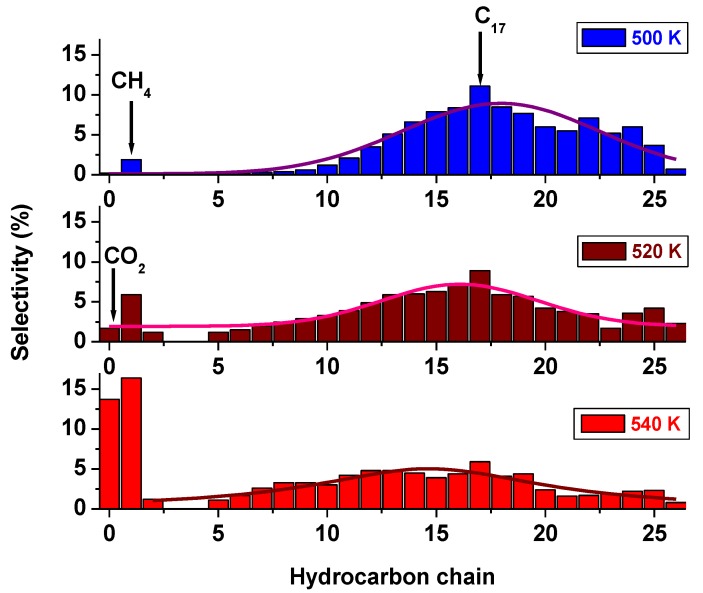
The selectivity plots of the Co/C catalyst tested at 500 K, 520 K and 540 K, 2 MPa pressure, and GHSV = 2000 cm^3^ h^−1^ g^−1^ of the catalyst.

**Figure 6:**
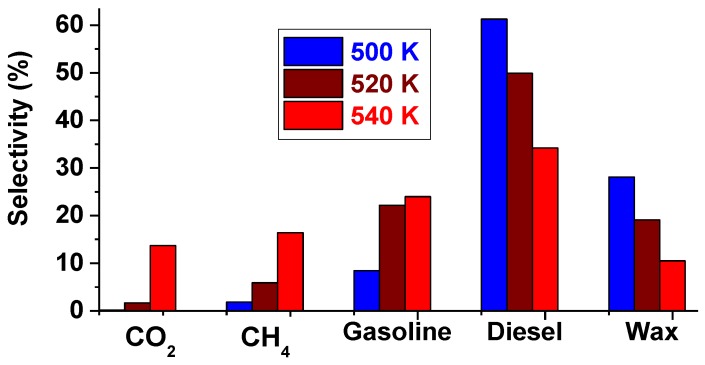
The aggregated product fractions of the Co/C catalyst tested at 500 K, 520 K, and 540 K (pressure = 2 MPa; GHSV = 2000 cm^3^ h^−1^ g^−1^ of the catalyst).

A pictorial is provided in Figure 5 to demonstrate the shift in the FTS product-distribution towards the left brought about by the influence of temperature, while Figure 6 shows their aggregated fractions. At 500 K, this catalyst produced only about 0.2% CO_2_ and 2% CH_4_, and approximately 2% CO_2_ and 6% CH_4_ at 520 K, but a whopping 14% CO_2_ and 16% CH_4_ at 540 K. On the other hand, at 520 K, the single-metal Fe/C sample produced 9% gasoline, 45% diesel, and 35% wax, while at 540 K, its selectivity was 24% towards gasoline, 31% diesel and 13% wax. 

**Figure 7:**
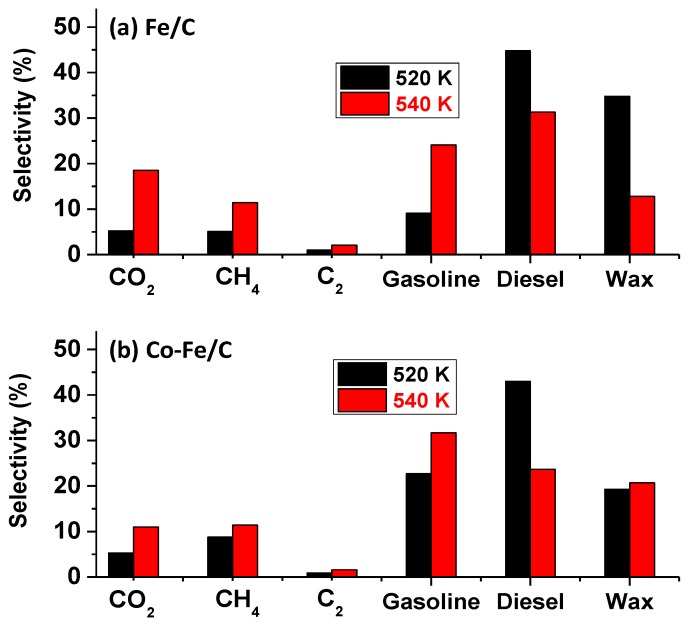
The aggregate selectivity of (**a**) Fe/C and (**b**) Co-Fe/C catalysts tested at 520 K and 540 K (pressure = 2 MPa; GHSV = 2000 cm^3^ h^−1^ g^−1^ of the catalyst).

Figure 7 depicts similar trends from the Fe-containing formulations, especially the Co-Fe/C bimetallic catalyst, which produced 23% gasoline, 43% diesel, and 19% wax at 520 K; and 32% gasoline, 24% diesel and 21% wax at 540 K, except that the Fe/C catalyst produced significantly more wax (35%) at 520 K than the bimetallic Co-Fe/C sample (19%). In addition, the Fe/C catalyst produced more CO_2_ (19%) than the bimetallic Co-Fe/C catalyst (11% CO_2_) at 540 K, probably due to the higher WGS activity, but both catalysts generated similar quantities of CH_4_ (11%). However, at 520 K, both Fe-containing catalysts showed equal selectivity towards CO_2_ (5%), but the bimetallic Co-Fe/C sample produced more CH_4_ (9%) than the Fe/C (5%).

#### 3.1.3. Alpha (α-Value) Determination

**Figure 8:**
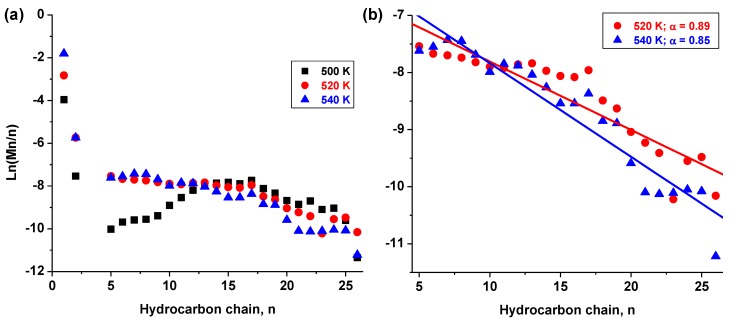
Modeling the ASF kinetics from selectivity data of the Co/C sample (**a**) at 500, 520, and 540 K, and (**b**) at 520 and 540 K.

From the catalyst selectivity and hydrocarbon distribution, kinetic data applying the Anderson–Schulz–Flory (ASF) model were used to determine the catalysts’ α–values. These are interpreted to be the relationship between the rate of chain propagation and the rate of chain termination. Usually the selectivity (Mn), obtained from mass or mol. % contributed by each hydrocarbon is plotted as natural log of (Mn/n) against the hydrocarbon distribution, (with n depicting the number of carbon atoms in each hydrocarbon chain). Figure 8 provides plots of the Co/C catalyst, with Figure 8a showing the full product distribution including CH_4_ at 500, 520 and 540 K. Since the sample tested at 500 K showed a significant deviation from the ASF model, this plot was isolated and Figure 8b shows the better fitting plots within the C_5_–C_26_ portion (at 520 K, α = 0.89, with a regression fitting, R^2^ of 0.84; and at 540 K, α = 0.85 with R^2^ = 0.90).

**Figure 9:**
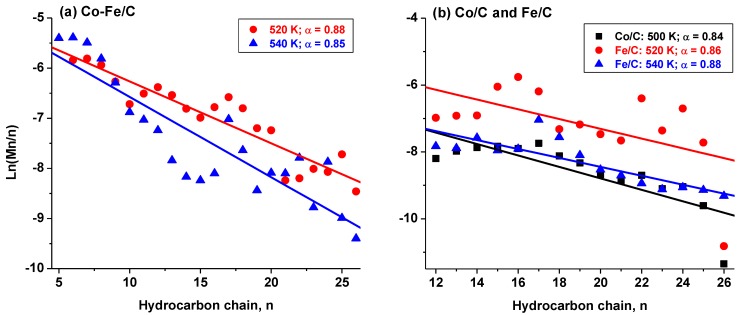
Modelling ASF kinetics using selectivity data of the (**a**) Co-Fe/C and (**b**) single-metal Co/C sample tested at 500 K, and the Fe/C sample tested at 520 K and 540 K.

The results of the catalysts with a less perfect fitting to the ASF model are given in Figure 9, where Figure 9a provides the α–values of the bimetallic Co-Fe/C sample tested at 520 K (α = 0.88 with R^2^ = 0.87) and at 540 K (α = 0.85, R^2^ = 0.79). Figure 9b represents results of the single-metal catalysts fitted in the range of C_12_–C_26_ in order to enhance the fitting, with the Co/C tested at 500 K (α = 0.84, R^2^ = 0.67), and Fe/C tested at 520 K (α = 0.86, R^2^ = 0.31), and 540 K (α = 0.88 with R^2^ = 0.72). Due to the fact that the catalysts’ selectivity data did not seem to conform to the typical linear ASF distribution, an attempt was made to estimate their α–values using the higher molecular weight hydrocarbons from n = 12. The catalysts’ estimated α–values were found to be in the range between 0.84–0.89, although with poor linear regression fits.

Table 1 summarizes all the raw data obtained for the catalyst performance in CO hydrogenation including their activity and selectivity, the probability of H_2_ utilization as indicated by the ratio of H_2_:CO uptake, as well as the estimated α–values with their corresponding linear regression data (R^2^). Since the ASF model favours production of the lighter hydrocarbons, formation of longer hydrocarbon chains in significant quantities will likely distort the pattern. In our earlier work, it was observed that various issues can affect conformity to the ASF model especially from poor solubility of the heavier components of the product stream. When some products fail to accumulate in the solvent during sampling, it can lead to a negative deviation from the α-values as predicted by the ASF model [47].

### 3.2. Catalyst Characterization

#### 3.2.1. BET Surface Area Analysis 

Catalyst characterization by BET analysis indicated that all the plasma-produced samples were almost identical, being both nanometric and non-porous in nature, as shown in Table 2. The BET specific surface area was in the range of 73–80 m^2^ g^−1^, and the average pore volume by Barrett-Joyner-Halenda (BJH) model was approximately 0.23 cm^3^ g^−1^, which principally arises from the interstitial volume of the nanometric carbon, while the average pore diameter (by 4V/A) was about 14 nm.

**Figure 10:**
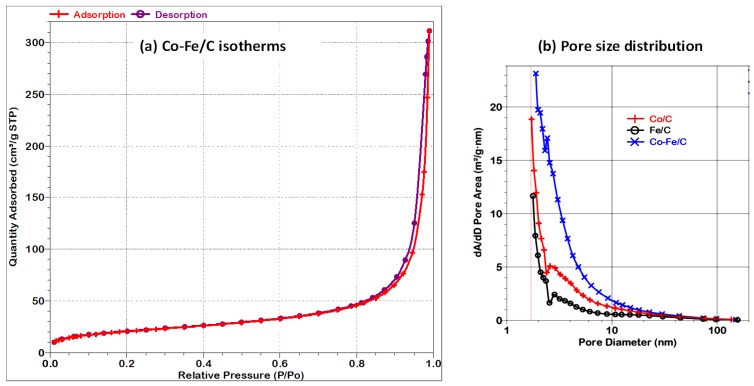
Plots showing (**a**) the adsorption-desorption isotherms of the fresh Co-Fe/C sample, and (**b**) the pore size distribution by the BET surface area analysis.

The porosity witnessed here could be associated with the packing of the nano-carbon powder particles since the superimposed isotherms (Type II) as indicated in Figure 10a shows that the samples are indeed non-porous [52]. Figure 10b provides the pore distribution plots proving that the samples are nanometric with limited micro-porosity (below 2 nm), while the augmented meso-porosity in the Co-Fe/C could be due to the larger metal nanoparticle size in the carbon support that subsequently creates sizeable interstitial voids in the nanomaterials.Some authors have found that the catalyst with BET specific surface areas of 40–60 m^2^ g^−1^ had similar average pore volumes in the range of 0.19–0.22 cm^3^ g^−1^, with the average pore diameter of about 14–22 nm [53]. This contrasts with other Fe-based FTS catalysts supported on spherical mesoporous carbon (SMC), exhibiting an order of magnitude higher in porosity, with a pore volume of 2.22 cm^3^ g^−1^ and a BET specific surface area of 767 m^2^ g^−1^ [54].

#### 3.2.2. Scanning Electron Microscopy (SEM) 

**Figure 11:**
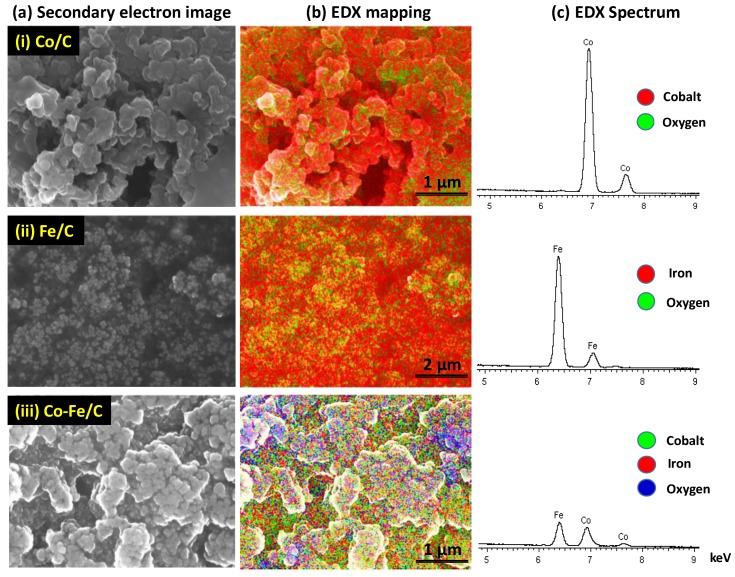
Sample SEM images of the used catalysts: (**i**) Co/C (**ii**) Fe/C and (**iii**) Co-Fe/C displaying (**a**) secondary electron images. (**b**) elemental EDX mapping, and (**c**) EDX spectra.

SEM imaging coupled with X-ray analytical methods (EDX mapping and spectroscopy) showed that all the catalysts (fresh and used) comprised uniformly dispersed metal moieties in the carbon support matrix as seen in Figure 11. Elemental analysis by X-ray mapping in the used catalysts confirmed that catalyst synthesis using plasma technology creates well-distributed metal nanoparticles in the materials, irrespective of the metal composition. The waxes generated during FTS were quite revealing as seen in the secondary electron images of Figure 11a.

#### 3.2.3. Transmission Electron Microscopy (TEM) 

**Figure 12:**
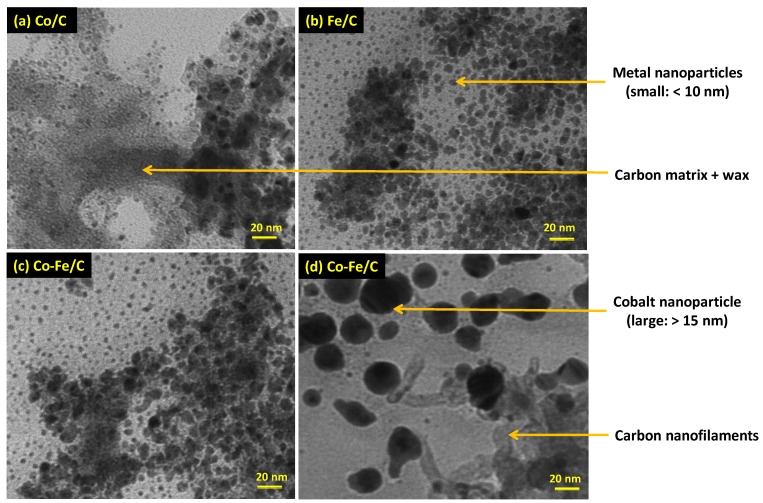
The TEM images of the used (**a**) Co/C (**b**) Fe/C and (**c**) Co-Fe/C catalysts with some sections having nanoparticles predominantly below ~5 nm, or ~10 nm, and (**d**) sections of the Co-Fe/C catalyst with larger nanoparticles ~20 nm and CNFs.

The analysis of 600 nanoparticles per sample by TEM imaging showed that both the used single metal Co/C and Fe/C catalysts had a bimodal distribution of small nanoparticles in the range below 5 nm and then at approximately 10 nm as shown in Figure 12a for the Co/C catalyst and Figure 12b for the Fe/C catalyst. The used bimetallic Co-Fe/C catalyst displayed a multi-modal nanoparticle distribution since certain catalyst sections were composed of nanoparticles predominantly below 5 nm, while other areas revealed nano-particles with a mean in the range of ~10 nm, as shown in Figure 12c. This occurrence was identical to that in single metal catalysts. 

**Figure 13:**
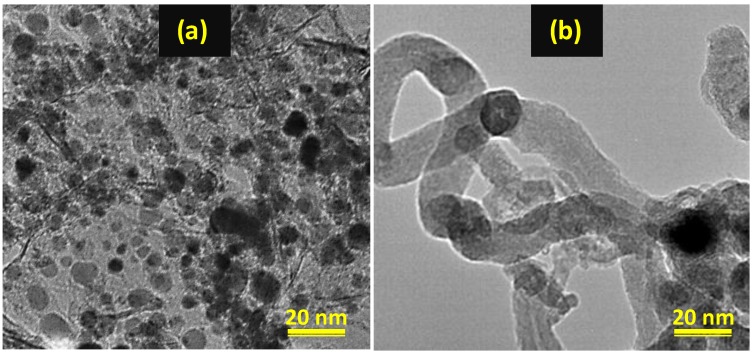
TEM images of the Co-Fe/C catalyst (**a**) freshly synthesized by plasma, and (**b**) after pre-treatment in CO, indicating formation of carbon nanofilaments (CNFs).

Additionally, the used Co-Fe/C bimetallic catalyst contained much larger nanoparticles, some of which were stretching beyond 20 nm, accompanied by the presence of carbon nanofilaments (CNFs) as seen in Figure 12d. Supplementary images are provided in Figure 13a for the freshly synthesized Co-Fe/C sample through plasma, contrasted with Figure 13b that represents the same, but used catalyst sample after FTS reaction where CO was used in the catalyst pre-treatment procedure. The presence of CNFs are unique to Fe-containing catalysts when reduced in CO, since a H_2_ reduction does not produce similar results. 

**Figure 14:**
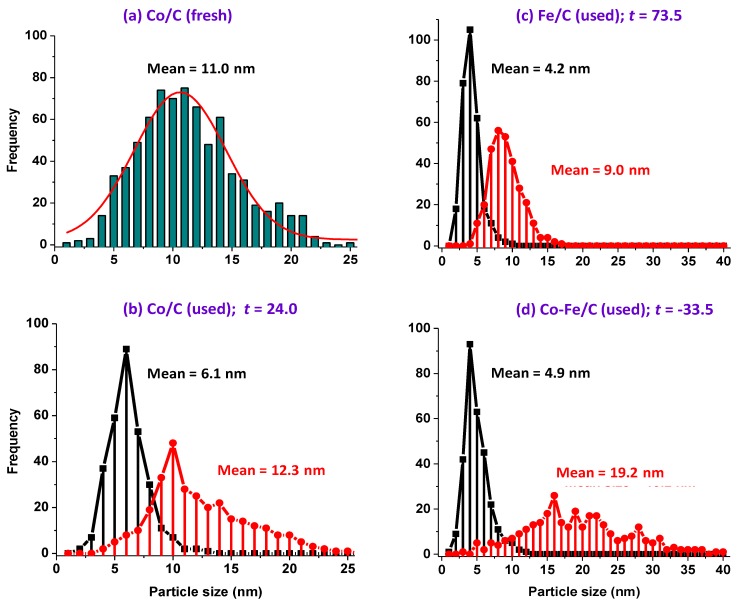
The particle size analysis by TEM imaging showing (**a**) a mono-modal nanoparticle distribution in the fresh Co/C catalyst [47]. (**b**) Bi-modal distribution in the used Co/C. (**c**) Bi-modal distribution in the Fe/C sample, and (**d**) multi-modal distribution in the used Co-Fe/C catalyst.

The *t*-test was therefore introduced to determine how significantly different these populations were from each other in each catalyst. It was observed that a freshly synthesized Co/C sample left the plasma reactor with a mono-modal particle-size distribution, as seen in Figure 14a [47], and the same particle-size distribution persisted in the used samples when pre-treated in H_2_ only [46]. However, by interrupting the H_2_ pre-treatment by a 10-h CO-reduction in-between, (as is the case in this work), a bimodal particle-size distribution emerged as portrayed by Figure 14b.

It was observed that the t-test for the used Co/C catalyst, which was reduced in both H_2_ and CO generated a t-value of 24, indicating that the two distributions were significantly different; with one population having a mean particle size of 6.1 nm, while the other one was 12.3 nm. Since plasma produces materials that are quenched before attaining equilibrium, this implies that strategically, plasma-derived catalyst materials can be manipulated to produce multiple morphologies during catalyst activation, alongside achieving desirable variations in activity and selectivity during the FTS reaction. These attributes can be utilized to modify catalyst performance. 

Similarly, the fresh Fe/C catalyst had a mono-modal nanoparticle-size distribution, but became bimodal after the FTS reaction, (or rather after CO-reduction), as seen in Figure 14c. This finding contrasts remarkably with our earlier work [46], whereby no major disparity was observed in the morphology and particle-size distribution of the fresh and used plasma-synthesized catalysts that were pre-treated in pure H_2_ only at the same temperature (673 K) for 24 h, the morphology and particle-size distribution did not vary much. However, in this study, the impact of the CO pre-treatment (10 h) was substantial, producing a clear variation in the particle-size distribution. Since one population of the used catalyst had a mean of 4.2 nm, while the other one had a mean of 9.0 nm, statistically, the two distributions were seen to be significantly different from each other, with a *t*-value of 74. Moreover, the used bimetallic Co-Fe/C catalyst showed a wide variety of features, ranging from a narrow distribution of nanoparticles with mean 4.9 nm to a broader distribution of nanoparticles ranging from 6.0 nm to 40 nm (mean = 19.2), interjected by the presence of CNFs. Figure 14d shows a *t*-value of −34 for the Co-Fe/C sample, and Table 3 summarizes all these findings, where s.d. is the standard deviation (σ).

#### 3.2.4. X-ray Photoelectron Spectroscopy (XPS) 

**Figure 15:**
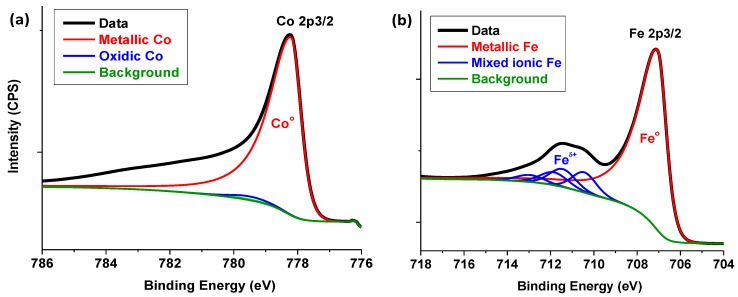
The XPS analysis of the fresh (**a**) Co/C and (**b**) Fe/C catalysts. Reproduced with permission from Reference [48]. Copyright Springer, 2018.

An XPS analysis of the fresh single metal catalysts (Co/C and Fe/C) shown in Figure 15a,b, respectively, has been published in an earlier article [48], and are only provided here for the sake of the reader and the completeness of the discussion. The chemical composition of these samples revealed that the predominant species in the Co-based catalyst was metallic (Co^0^) with virtually no oxides detected, while the Fe/C sample comprised both the metallic (Fe^0^) species and the mixed ionic species (Fe^2+^/Fe^3+^) on the surface of the material. 

**Figure 16:**
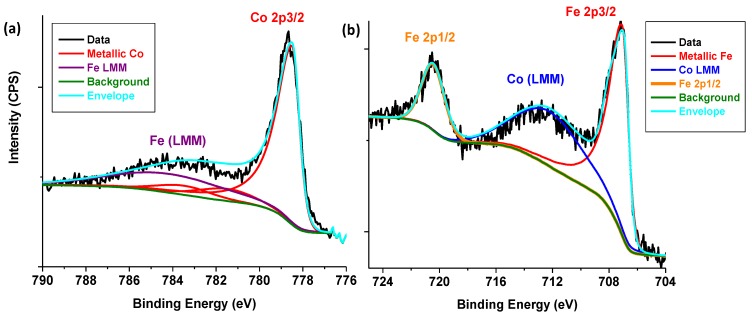
The XPS analysis of (**a**) Co metal, and (**b**) Fe metal in the fresh Co-Fe/C catalyst samples collected from the main plasma reactor.

XPS results of the 50%Co-50%Fe/C bimetallic catalyst are presented here for the first time and the intense asymmetric Co 2p3/2 peak at 779 eV shown in Figure 16a signify and confirm the presence of the metallic (Co^0^) species. In close proximity is the broad Auger peak at 784 eV for metallic Fe (LMM). On the other hand, the intense Fe 2p3/2 peak at 707 eV in Figure 16b identified the metallic Fe^0^ species, while the metallic Co (LMM) Auger peak at around 712 eV was sandwiched between the metallic Fe 2p3/2 at 707 eV and the Fe 2p1/2 at 721 eV. The standard deviation (s.d.) of fitting the envelope to the data was 0.9382, but 0.9379 when the fit included a carbide (Fe_3_C) peak at 708 eV, which made the presence of carbides in the samples doubtful since the difference in the s.d. was practically inconsequential.

**Figure 17:**
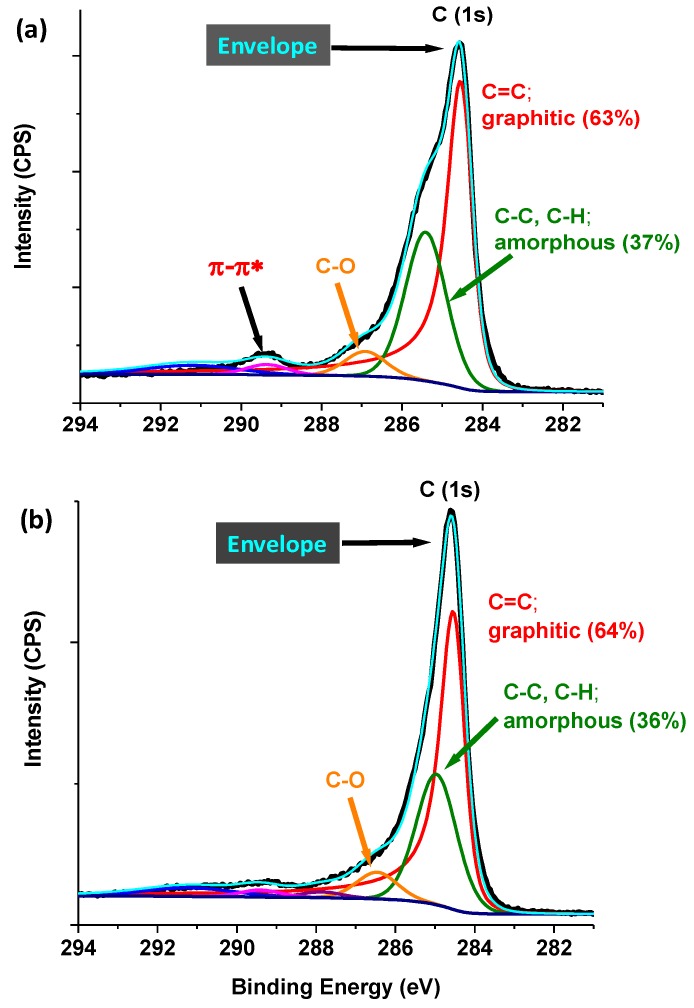
The XPS plots quantifying the C-support in the fresh Co-Fe/C catalyst samples collected from (**a**) the main plasma reactor and (**b**) the secondary plasma reactor.

In observing the C-peak with a binding energy of 284 eV in the bimetallic Co-Fe/C catalyst, two different types of carbon were found in the catalyst, and when quantified as shown in Figure 17, the support was predominantly graphitic-C (G), which comprised about 63–64% and partly amorphous or disordered-C (D) with 36–37%. The G-component was confirmed by the (π → π*) transition peak displayed at about 289 eV, seen in Figure 17a, although a bit diminished in Figure 17b.

#### 3.2.5. X-ray Diffraction (XRD) 

**Figure 18:**
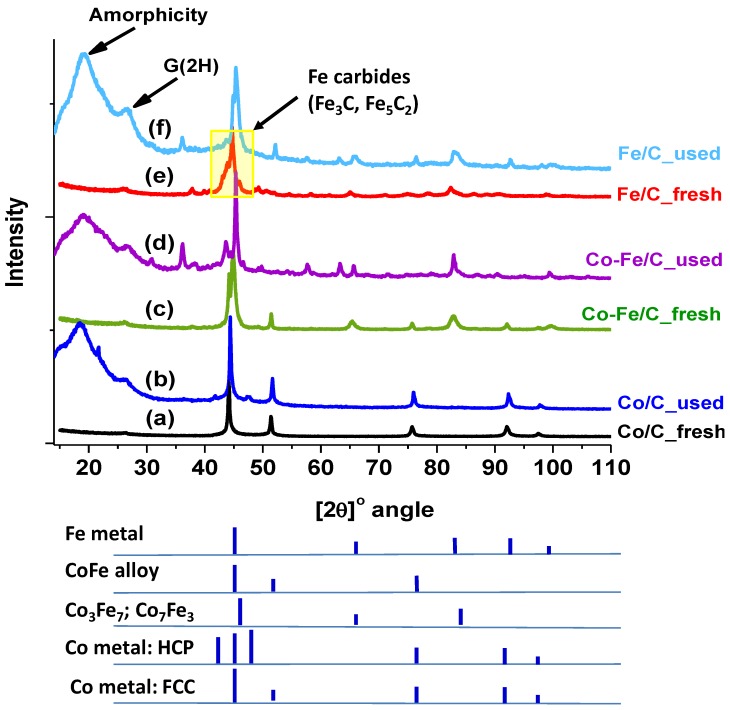
The XRD patterns of the catalyst samples for (**a**) fresh Co/C (**b**) used Co/C (**c**) fresh Co-Fe/C, (**d**) used Co-Fe/C (**e**) fresh Fe/C and (**f**) used Fe/C.

Several unique challenges exist with XRD analysis of our samples because the isolation of used catalysts from the FTS products is difficult and it prevents proper sample analysis. For example, cleaning the sample from the FTS oils and waxes oxidizes it immediately because the plasma-synthesized samples are potentially pyrophoric and cleaning the samples defeats the purpose of the analysis. Consequently, oily slurries from the FTS reactor were used in the XRD analyses. Figure 18a,c,e indicate the XRD patterns of the freshly synthesized catalyst samples. However, huge broad peaks appear in the XRD spectra of the used samples below a 30° [2θ]-angle, as shown in Figure 18b,d,f, indicating that the samples are highly amorphous, which is not necessarily true. All the used samples largely contained the G(2H)-phase, graphite of the hexagonal crystal structure. This means that during gasification, the D-phase was most likely eliminated. 

It has been advanced that the chemical versatility of carbon, especially its high catenation power (to form longer chains and structures) enhances its capacity for sp^2^ to sp^3^ hybridization and extraordinarily binds to other atoms [2]. In view of this, we suspect that sp-orbital hybridization is the main reason why the metal moieties in the materials get encapsulated by G, in addition to causing the growth of CNFs.

**Figure 19:**
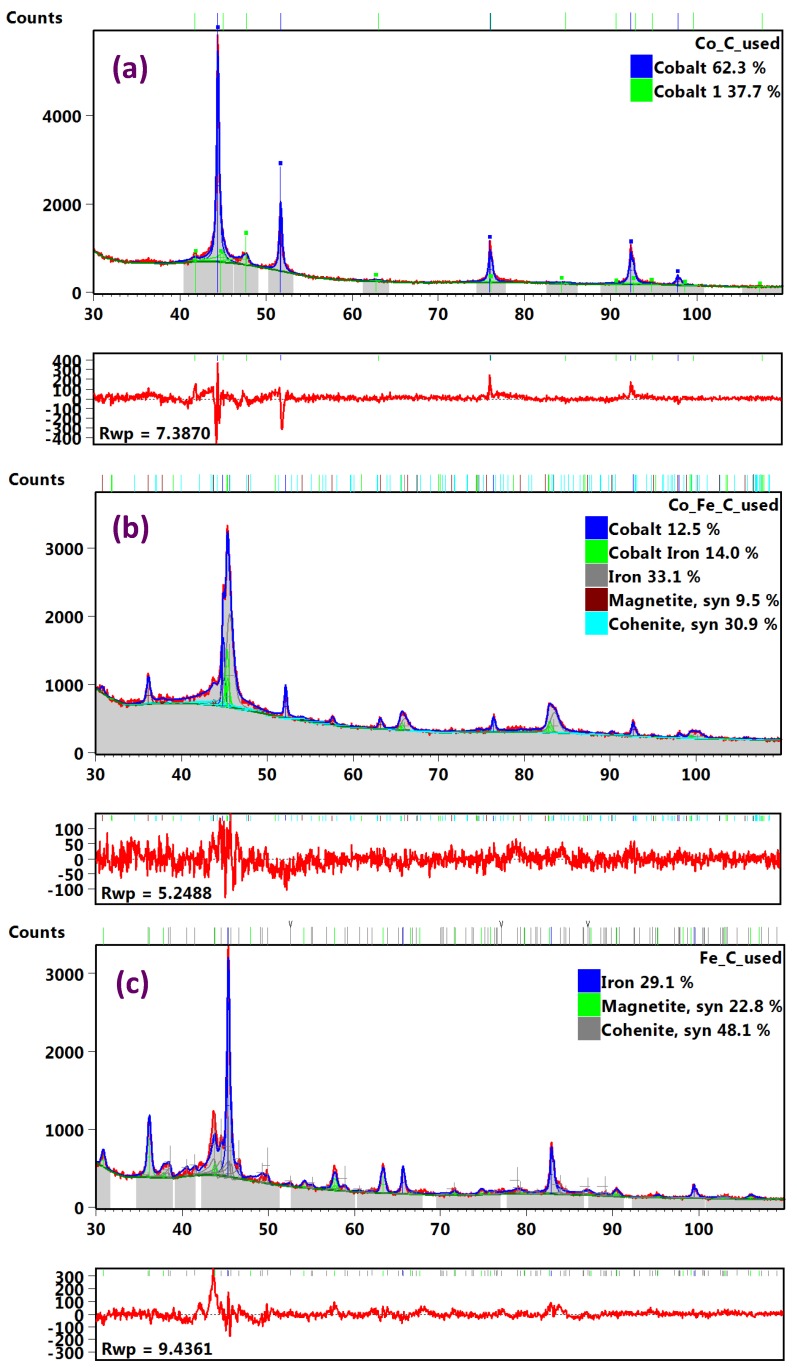
The RQA curve fitting of the used (**a**) Co/C (**b**) Co-Fe/C, and (**c**) Fe/C catalyst samples by XRD analysis.

Nonetheless, since some significant differences were conspicuous in the XRD patterns of the fresh and used samples, phase quantification by RQA was attempted, with peak fitting done only for the phases containing Co and Fe, while excluding carbon’s G(2H) and the D peaks appearing below 30° [2θ]-angle. After eliminating all amorphicity, Figure 19a–c depict the XRD patterns and their residuals arising from the RQA curve fittings for the used Co/C, Co-Fe/C bimetallic and Fe/C samples, respectively.

Table 4 summarizes the RQA results showing that the single-metal catalysts contained a significant amount of metallic species, where the Co/C sample composed a predominantly face-centered cubic (FCC) crystal structure (62.3%) and some hexagonal closed packed (HCP) crystal structure (37.7%). Both phases appeared in the used sample in the same ratio as the original fresh Co metal that was injected into the plasma, having comprised two phases (62% FCC, and 38% HCP) as analyzed earlier by RQA [45]. In another previous study, it was evident that the HCP phase was vanquished by plasma as observed from both the fresh catalyst and the used sample after pre-treatment in H_2_ alone [47]. However, in this work, the HCP phase seems to be regenerated in the used sample after exposure to a CO reduction. 

The Fe/C sample on the other hand, contained mainly the α–Fe phase of metallic iron (29%) having the FCC crystal structure. Besides, carbide and oxide phases were identified in the used Fe/C sample (48% Fe_3_C and 23% Fe_3_O_4_; magnetite), both of which were also found in the used bimetallic Co-Fe/C catalyst (31% Fe_3_C and 10% Fe_3_O_4_). Moreover, the Co-Fe/C catalyst contained the following pure metallic phases: the Co^0^ (13% FCC) and α–Fe^0^ (33% FCC) crystal structures. There were also nano-alloy structures chiefly of the Fe_3_Co form detected in the bimetallic sample (14%).

## 4. Discussion

### 4.1. Influence of Pre-Treatment Procedure on Catalyst Activity

A comparative study has been conducted for Fischer–Tropsch catalysis using three different plasma-synthesized catalyst formulations supported on carbon (that is, Co/C, Fe/C and the Co-Fe/C). To contextualize the current investigation, earlier studies showed that in order to expose the reactants to the metal nanoparticles in plasma-synthesized catalysts to the reactants, some of the excess C-matrix must be extracted by gasifying the C into CH_4_ and other gaseous products [55]. However, in some cases, the over-reduction, particularly of the Fe-based samples, was observed to lower catalytic activity as it ruined the carbidic phase, which is deemed to be the active species in FTS; and incidentally, it was found that the plasma-synthesized Fe catalyst was more sensitive to the reduction procedure (e.g., temperature, time) than the Co-based catalyst [46]. 

Moreover, when samples were pre-treated in pure H_2_ alone (e.g., Co/C at 673 K), the catalyst was observed to deactivate more rapidly with TOS than the samples reduced in CO followed by a H_2_ reduction [52]. Furthermore, the samples that were pre-treated in CO followed by a H_2_ reduction were observed to be comparatively more effective in CO hydrogenation than those reduced in H_2_ alone since a CO-reduction step limited the production of CO_2_ as a side FTS product. The CO-reduction step in catalyst pre-treatment has been shown to produce a more stable catalyst over TOS [52], accompanied by a lower H_2_O production [56]. 

In the current investigation, a 3-step reduction was initiated: the first H_2_ reduction was to gasify the excess C-matrix around the metal nanoparticles, followed by carburization using a CO-reduction step to activate the catalysts towards a higher selectivity for FTS products, especially in the Fe-based catalysts on order to produce the metal-carbide phase. The final H_2_ reduction was to ensure that any possible C-deposits created during CO-reduction were also gasified, while any Co carbides formed in the samples were converted back into the active metallic form [57]. In short, since we know from past studies that H_2_-reduction does not affect the morphology of the catalyst [46], this protocol can be viewed as a 44-h H_2_-reduction interrupted by a 10-h introduction of CO in the reducing medium after 24 h. It is therefore assumed that higher catalytic activity and the various morphologies generated in the used catalysts came about as a result of the carburization with CO. 

Reduction with H_2_ at 673 K has been shown to restore the catalyst activity, particularly from depositions of “free” carbon, although bulk-phase carbides existing at temperatures below 550 K could also caused a marked decrease in activity [58]. From Figure 1 and Figure 2, it was observed that after each 24-h experimental run, the samples were still active at every operational temperature, with very little signs of deactivation. The possible deactivation is attributed to the increasing H_2_O vapor pressure in the system. This, therefore, means that the carburization step in combination with the H_2_-reduction cycles promotes the production of active and stable catalysts online, besides being highly selective for CO hydrogenation towards the formation of C_5+_ fraction.

### 4.2. Temperature Effect on Hydrogen Utilization Efficiency in CO hydrogenation

Earlier work with the 50%Co-50%Fe/C formulation indicated that the catalyst produces less H_2_O in FTS (at 533 K, 2 MPa, and H_2_:CO = 2.0) when reduced in CO only than when reduced in H_2_ only [56]. Recent work with the Co/C catalyst showed that successive reductions in CO followed by H_2_ improves the catalyst performance (at 518 K, 2.2 MPa, and H_2_:CO = 1.7), producing relatively less H_2_O when compared to the sample that was reduced in H_2_ only [52]. In this work, both the above-named catalysts (Co/C, Co-Fe/C) in addition to the Fe/C one have been reduced successively, first in pure H_2_, then in pure CO and finally in pure H_2_ again. Consequently, a higher degree of H_2_-incorporation into the FTS products was evident from these samples at lower FTS temperatures, and an increase in temperature produced a gradual decrease in the H_2_:CO uptake ratio. It was observed that a significant quantity of the converted CO formed either CO_2_ or CH_4_ at the higher temperatures, leading to poor CO hydrogenation towards the C_5+_ fraction. 

Some authors have shown that increasing FTS reaction temperature lowers the probability of hydrocarbon growth and, hence, the catalyst’s α-value. This means lower C_5+_ selectivity and higher CH_4_ production [59], mainly due to higher products desorption rates and lower molecules’ residence time on the catalyst surface. Therefore, in agreement, this work shows that at 540 K, the product spectrum was richer in gasoline fraction due to the predominant presence of shorter hydrocarbons. In order to determine the efficiency of the H_2_-incorporation in FTS products, the amount of H_2_O generated over a period of 24 h in each testing cycle was measured and the H_2_ traced back to its origin using Equations (4) and (6). The method used is outlined in a previous manuscript [60], where it was presumed that there was a direct relationship between the amount of H_2_O produced and the degree of CO hydrogenation. A summary of these results is provided in Table 5. 

For example, take the case where 14 cm^3^ of H_2_O was generated by the Co/C catalyst at 500 K. From the selectivity data, which provided the FTS product spectrum, 15.6% of the total H_2_ feedstock was utilized in producing C_5+_ while only 0.5% was incorporated in CH_4_. This means that for every CH_4_ molecule produced, about 35 hydrocarbon monomers, –[CH_2_]– were formed; i.e., (C_5+_):(CH_4_) = 35. Thus, in terms of useful product selectivity, although the catalyst activity was relatively low (at ~18% CO conversion), these are proven to be the most efficient FTS reaction conditions with this catalyst. Both the single-metal Co/C and Fe/C catalysts at 520 K were operating at the same H_2_ utilization efficiency (~11). The least efficient operation was with the same Co/C catalyst at 540 K; at these conditions, despite the high CO conversion (85%), for every CH_4_ molecule produced, only three –[CH_2_]– monomers were formed towards the (C_5+_) hydrocarbon chains. Additionally, these findings imply that the feedstock ratio of H_2_:CO = 2.2 was not H_2_-efficient at higher temperatures. Studies have shown that increasing the H_2_:CO feed ratio decreases the catalyst’s α-value because a high H_2_ concentration promotes chain-growth termination, which leads to increased CH_4_ production and decreased C_5+_ selectivity [59]. 

Bearing in mind that the original intention of this study was to test the catalysts for HT-FTS, which normally operates above 600 K by gradually increasing the temperature from 500 K, this objective fell short. The reason being by 540 K, the H_2_ utilization efficiency for CO hydrogenation was fairly poor, with large quantities of both the CO and H_2_ in the feedstock gas going into CO_2_ and CH_4_, both of which are undesirable in FTS. This observation led to the conclusion that these catalysts are all designed to operate, typically, below 520 K; that is, within the LT-FTS regime only. No further tests were performed beyond 540 K. This was surprising because Fe is known to be a very good HT-FTS catalyst.

### 4.3. Catalyst Characterization

Since the catalysts produced through plasma have high proportions of graphitic carbon, elemental analysis by conventional methods such as the inductively-coupled plasma mass spectrometry (ICP-MS) are not applicable because it is difficult to grind and completely dissolve G. Nevertheless, an elemental analysis of the single metal catalysts (Co/C and Fe/C) was done by total carbon ignition and found to be roughly 25% atomic % mass-by-mass (at % metal:carbon) [46]. This was confirmed by SEM analysis using EDX semi-quantitative analysis, which indicated a value of 25% (±5). EDX mapping showed that the samples had a homogeneous distribution of the metal moieties in the C-support. The uniform metal dispersion onserved without any indication of metal particle segregation attested attests to the robust nature of the SPS technology in producing high-quality nano-catalyst materials. Catalytic nanoparticles have been shown to be highly reactive [61] and the substantial change in the catalyst morphology and particle size distribution after pre-treatment in CO as indicated by TEM analysis implies that plasma-synthesized materials have a great capacity to be manipulated through different reaction conditions to produce certain desired outcomes in FTS catalysis. 

This study has shown that plasma produces metal nanoparticles having a Gaussian-type of particle size distribution with a mean of ~10 nm. However, carburization with CO creates a mechanism that produces bimodal distribution plots in the single metal catalysts. This mechanism is currently not well understood, but it could be related to metal nanoparticles entrapment in the graphitic carbon. Some authors have also succeeded in producing nano-sized FTS catalysts with bimodal pore distribution, where the smaller pores serve as traps for Co nanoparticles, preventing sintering and agglomeration due to confinement effects [62]. A similar phenomenon has been reported for the Fe-based materials where enhanced FTS activity and stability has been recorded through the preferential formation of bimodal crystallite sizes of Fe nanoparticles in bimodal mesoporous structures having average pore sizes of 3.6 and 5.4 nm [63]. Since we noticed an increased concentration of the smaller nanoparticles in our samples (with a mean size of ~5 nm), catalytic activity was also observed to be high. It is suspected that the smaller particles are more active, and therefore, the production of smaller nanoparticles is essential in FTS because they derive their reactivity from the large number of coordinatively unsaturated atoms relative to the total number of metal atoms in their crystallites. It is well known that surface atoms located at the steps, corners, and edges of nanoparticles exhibit the highest catalytic activity due to their low coordination number. 

Further, TEM imaging indicated that the bimetallic Co-Fe catalyst had both small (5 nm) and large (10 nm) nanoparticles, besides showing a wide distribution of even bigger nanoparticles including CNFs. In earlier work, we showed that the CO-reduced Co/C catalyst did not display the presence of CNFs [52], and the same has been confirmed in this study. Further, another previous study had indicated the presence of CNFs in all the CO-reduced Co-Fe/C catalysts [56], which is again confirmed in the current work. Therefore, only the Fe species seem to have the capacity to produce CNFs over these catalysts under the prescribed reaction conditions.

A semi-quantitative analysis of the C-support of the fresh catalysts by XPS identified the G-form (~64%) as the predominant phase and that some D-phase comprises ~36%, as shown in Table 6. In a recent publication, we showed that larger metal nanoparticles with a mean size of ~11 nm (compared to ~9 nm) form more D-phase and, hence, have a lower BET specific surface area, because surface area depends on the amount of G-phase in the sample [48]. In the current scenario, the particle size of the fresh Co-Fe/C sample is relatively much larger (14 nm), with a diminished quantity of the G-phase (64%) and, therefore, its BET specific surface area would be expected to be much less, and yet it has the greatest surface area amongst the three. The contradiction may arise due to the fact that the packing of the nano-carbon support particles in the bimetallic Co-Fe/C catalyst seems to create more mesoporous (2–50 nm) spacing than with the single metal catalysts as shown in Figure 10, although their surface areas are within the same range (76 ± 3 m^2^ g^−1^).

In terms of their performance in FTS, all the three catalysts were close at 500 K and, in fact, the Co-Fe bimetallic formulation showed the highest CO conversion at 520 K (with 57%) as compared to the other single metals (46% for Co/C and 37% for Fe/C). When reduced in H_2_ alone, it was observed that the 50%Co-50%Fe/C bimetallic was completely inactive at 493 K, while the Co-rich samples (Co/C, and 80%Co-20%Fe) were more active. The Co/C was the most active with over 40% CO conversion [56]. Some authors have observed that in bimetallic formulations, the alloy with an intermetallic ratio of Co:Fe = 1 is not a critical component in enhancing both the FTS activity and selectivity of the carbon-supported catalyst, but rather a Co-rich combination being the most significant, with a metal ratio close to Co_2_Fe [64]. However, in the current study, it is shown that in spite of catalyst composition, a CO-reduction step alters the activity of the materials substantially.

From the RQA-XRD analysis, the pronounced presence of the HCP crystal structure (38%) in the used Co/C sample is an interesting observation because HCP as the active phase in the Co catalyst has been shown to exhibit greater intrinsic activity than the FCC [65]. This means that the CO-reduction increased the chance of generating the HCP phase when compared to H_2_-reduction. Some authors state that the selective formation of metallic Co in the HCP crystal structure through successive carburization and hydrogenation of a used catalyst is the key to both improved catalytic activity and the effective in situ regeneration [57], as summarized in Equation (8).

(8) $$\;{\;\mathrm{CoO}}_{\left(\mathrm{FCC}\right)}\overset{\mathrm{CO}}{\to }{\;\mathrm{Co}}_{2}C\;\overset{H_{2}}{\to }{\;\mathrm{Co}}_{\left(\mathrm{HCP}\right)}\;$$

Furthermore, from the XRD analysis, Fe-containing samples were observed to have a concomitant presence of carbide and oxide species in significant quantities. Some authors think that the formation of non-stoichiometric iron-oxide-carbide species which though relatively less stable, is an important combination in Fe-based FTS catalysis [66], and they consider it to be more active and more selective towards olefin formation than the known χ-Fe_5_C_2_ carbide. This implies that total reduction of the catalyst to metallic state or pure Fe carbides is not beneficial to the FTS process. Our XRD analysis shows that there were rather many phases and probably various active species that were formed either during the plasma synthesis or during CO reduction. Since it was apparent that the catalyst samples did not contain homogeneous active species, it implies that the materials could be applying different FTS reaction mechanisms simultaneously and for that reason, the product spectra did not conform to the linear ASF kinetics. Overall, there are three main interrelated factors in this investigation that could cause the FTS product distributions not fit the usual ASF kinetics:

(i) *Solvent effect*: since some of our earlier works have produced results conforming to the ASF model, the tests had been conducted in hexadecane (C_16_) solvent, but in this study, squalene (C_30_) was used instead. It has been argued that when a significant portion of the heavier FTS product components fail to dissolve in the solvent, it lowers its amount in the sample drawn for analysis and this may distort the linearity of the ASF plot [47]. In addition, if polar products such as alcohols are in high proportions, they too may fail to dissolve in the organic medium of the liquid phase. Since C_17_ was the most intense peak, it could be perceived as though the catalysts were most selective towards the production of C_17_, or that the other products were less soluble in the current solvent.
(ii) *CO-reduction effect on the catalysts*: previous studies with H_2_-reduced catalysts indicated near linear plots that conform to the ASF model [47]. However, in this work, the introduction of CO reduction in the catalyst pre-treatment procedure was observed to create a myriad of metal particles and carbon support with different sizes and morphologies, ranging from single-metal zero-valent particles, to metal carbides, bimetallic nano-alloys and carbon nanofilaments. Each one of these moieties in the catalyst could impact the FTS reaction differently.
(iii) *Metal nanoparticle-size effect*: TEM imaging showed that the multi-modal metal nanoparticle-size distributions were generated by CO reduction, and these results were significantly different from those of H_2_-reduced catalysts, which showed mono-modal (near Gaussian-type) nanoparticle size distribution. It is suspected that having a substantial variation in the particle-size distribution created energetically diverse active sites, leading to different reaction paths and mechanisms in FTS activity and hence poor conformity to ASF kinetics, which require energetically homogenous active sites.

TEM analysis showed a multi-modal distribution of the metal nanoparticles in the three catalysts. In the literature [67], it has been shown that (i) crystallite size impacts FTS activity for Co crystallites smaller than 10 nm, and (ii) increasing the Co crystallite size leads to higher CO hydrogenation towards C_5+_ selectivity, extending past the 6–8 nm region. Our tested catalysts, which contain a high percentage of nanoparticles below 5 nm were highly active for FTS. Obtaining C-supported FTS catalysts with extremely small nanoparticles is not unusual; some authors have achieved Co nanoparticles with 2–6 nm (TEM analysis), using the microemulsion method, and the nanoparticles are mostly confined inside the CNTs [68]. In this work, small metal nanoparticles have been achieved through plasma synthesis and CO reduction without the use of CNTs, because the nanoparticles are confined in the G-support matrix.

Moreover, the high H_2_:CO ratio of 2.2 used in this study could have contributed to the high catalytic activity because some authors have linked catalyst reactivity to H_2_ exposure since CO molecules dissociate more efficiently on the larger Co nanoparticles (15 nm) than on the smaller ones (4 nm), and higher exposure of Co nanoparticles to H_2_ has been found to enhance CO dissociation rates [69]. Therefore, since higher H_2_ concentration tends to favor chain termination reactions, it leads to the formation of shorter-chain hydrocarbons, which enrich the gasoline fraction [70]. Optimally, it has been observed that an FTS catalyst must have metal nanoparticles within a narrow range of about 6–8 nm [71], and this assertion has been supported by kinetic studies indicating that the FCC structure of Co metal favors the H-assisted CO dissociation mechanism [72], and we find our samples to be highly dominated by the presence of FCC crystal structures.

### 4.4. Benefits of Using Plasma Technology in Synthesizing FTS Catalysts

In Fe-catalyzed FTS, the reaction mechanism depends on the presence of Fe carbides [66], and currently in industry, the Fe carbides are generated mainly by carburizing the Fe oxides with CO [73]. In this work, the carbides were produced in plasma. Earlier, we attempted to benchmark our plasma-synthesized Fe-based catalyst with the commercial nano-hematite (Fe-NanoCat^®^) by carburizing the nano-hematite using CO. Although the Fe-NanoCat^®^ catalyst was still very active for CO hydrogenation in FTS, it showed excessive nano-particle agglomeration, while the plasma-derived samples did not [46]. On the other hand, metallic Co, which is the predominant phase in our samples is considered to be the most active FTS species in Co-based catalysts [65], and these too were generated through plasma. 

Therefore, the benefits of plasma technology in regard to catalyst synthesis over other conventional methods such as precipitation or impregnation methods, include the following:

- *SPS technology shrinks synthesis steps*: Since plasma technology is a single-step method, it diminishes the number of operational factors and repetitive control parameters involved at each stage (e.g., synthesis pressure, temperature, pH, time, purity), besides lowering the labor and materials costs, which makes catalyst production process much easier, and this greatly increases the probability of reproducing the material [45].
- *Plasma synthesis is a robust and adaptable method*: Plasma produces high-quality catalysts, which are both nanometric and non-porous in nature as revealed by BET surface area measurements. From a microscopic (SEM) analysis using EDX mapping, the materials show high metal particle dispersion with uniform distribution in the carbon matrix and all the samples are remarkably identical in morphology in spite of their compositions [60]. Moreover, SPS technology provides such versatility that one recipe can be used to produce a variety of catalytic formulations and this makes the synthesis method highly reliable [43].
- *Plasma fosters the design of functional nanomaterials*: Since the FTS reaction involves the production of a mixture of large polymeric molecules such as waxes that easily cause catalyst deactivation, the nanometric and non-porous nature of these materials make them ideal for circumventing mass transfer and diffusion limitations during FTS.
- *Production of ready-to-use catalysts*: Catalysts produced through plasma do not require elaborate improvement procedures or sophisticated pre-treatment methods before their application in the FTS process, and can be promoted with other metals both during production [60], and after plasma synthesis [74]. In this work, we show that identical materials can be modified through strategic pre-treatments in order to produce a diversity of morphologies and by varying the reaction conditions, different FTS products can be obtained.
- *In situ production of graphitic carbon support*: With SPS technology, the metallic active phases (Co^0^ for Co-based catalysts and Fe_x_C for Fe-based catalysts) are produced concomitantly with the C-support in the plasma [46]. This contrasts with traditional approaches where if a C-support is utilized, for example, activated carbon, carbon nanotubes or CNFs [75], the support must be produced in another process first before metal deposition.
- *Superior catalytic performance*: In this work, the catalysts did not show many signs of deactivation after 24 h of FTS. In earlier works, catalysts produced through plasma showed superior catalytic performance (~4 times more active) when compared to those prepared by precipitation or impregnation methods under identical FTS reaction conditions [45]. Plasma-synthesized metal nanoparticles do not seem to agglomerate during the FTS reaction like catalysts prepared by precipitation or impregnation methods when subjected to high-temperature treatment [46]. Besides, the catalysts do not deactivate due to carburization when reduced in CO [56]. In fact, this work proves that CO reduction has a positive effect on catalytic performance.

## 5. Conclusions

The results of a comparative study for three catalysts (single-metal Co/C, Fe/C and the bimetallic 50%Co-50%Fe/C) are presented. The catalysts were synthesized through plasma and with a BET specific surface area of ~80 m^2^.g^−1^; they are remarkably identical in morphology too (as seen from SEM imaging coupled with EDX elemental mapping). They show a high metal particle dispersion and a uniform distribution since the metal nanoparticles do not show any segregation in the carbon matrix. The catalysts were tested on bench-scale for the production of synthetic automobile fuels in typical FTS process conditions (500–540 K; 2 MPa pressure; H_2_:CO = 2.2; GHSV = 2000 cm^3^ h^−1^ g^−1^ of the catalyst).

XRD analysis revealed the presence of Co^0^ with an FCC and HCP crystal structure (62% and 38% respectively) in the used Co/C sample, while cohenite (Fe_3_C) and magnetite (Fe_3_O_4_) phases were evident in the used Fe/C and bimetallic Co-Fe samples. The FCC metallic phase (α–Fe) dominated in both the fresh and used Fe-containing samples, which was confirmed by XPS analysis. Semi-quantitative analysis of fresh catalysts by XPS showed that the carbon support matrix was ~65% G and ~35% D. Further, TEM imaging revealed that the metal nanoparticles do not sinter even when exposed to high-temperature treatments (in excess of 673 K) before and during the FTS reaction; the main reason being that the nanoparticles are “caged” in the graphitic-C framework, which prevents particle interaction and agglomeration. Moreover, the catalysts did not seem to deactivate as a result of carburization or oxidation as shown through XPS and XRD analyses.

Catalyst modification by consecutive reduction at 673 K using H_2_ (24 h), CO (10 h), and then H_2_ again (10 h), activated and improved the FTS activity remarkably. This yielded catalyst stability and high selectivity for CO hydrogenation over TOS, but with low conformity to the Anderson–Schulz–Flory (ASF) distribution. Although having low reliability due to imperfect linear regression fittings, the estimated α–values in this study were in the range of 0.84–0.89. However, earlier works had shown similar values, but with much higher linear regression fittings. Reduced conformity to the ASF distribution could be attributed to several interrelated issues including: (i) solvent effects, where a large portion of the product stream fails to dissolve in the liquid phase for analysis; (ii) CO-reduction effect on the catalyst, which generates substantial variation in the metal nanoparticle-size distribution; and (iii) metal particle-size effect, which lead to the creation of energetically diverse active sites, and since ASF kinetics require energetically homogenous active sites, catalyst samples in this work may have fashioned several different reaction paths and mechanisms. 

Generally, higher temperatures enhanced the yield of the gasoline fraction, accompanied by a decrease in hydrogen utilization efficiency (lower H_2_:CO uptake ratio). On the other hand, lower temperatures enriched the diesel fraction, with a better hydrogen utilization efficiency since a higher H_2_ percentage was incorporated in C_5+_ molecules. The Co/C catalyst gave up to 98% C_5+_ selectivity at 500 K (Table 5). An attempt to raise the FTS temperature beyond 540 K generated excessive CO_2_ and CH_4_, both of which are undesirable because they lowered the C_5+_ selectivity to ~70–80%. It was observed, therefore, that the catalysts are better fitted for LT-FTS operations. TEM imaging showed bimodal nanoparticle size distribution in the used catalysts after CO reduction possibly by a reaction mechanism that increases the number of smaller nanoparticles with mean size of ~4–6 nm. The smaller nanoparticles are presumed to be highly energetic and, therefore, catalytically more active than the original plasma-synthesized nanoparticles with mean size of ~9–11 nm.

## Figures and Tables

**Table 1:** The summarized performance data of the Co/C, Fe/C and 50%Co-50%Fe/C catalysts tested at various temperatures using 2 MPa pressure, and GHSV = 2000 cm^3^ h^−1^ g^−1^ of the catalyst.

Catalyst	T (K)	Conversion (mol. %)			Selectivity (mol. % C)						ASF Model	
		H_2_	CO	H_2_:CO Ratio	CO_2_	CH_4_	C_2_–C_4_	C_5_–C_12_	C_13_–C_20_	C_21+_	α-Value	Fit (R^2^)
**Co/C**	500	25.2	17.5	1.5	0.2	1.9	0.2	8.4	61.3	28.1	0.84	0.67
	520	53.4	46.2	1.2	1.7	5.9	0.4	22.2	49.9	19.1	0.89	0.84
	540	76.9	85.0	0.9	13.7	16.4	1.2	24.0	34.2	10.5	0.85	0.90
**Co-Fe/C**	500	19.5	15.8	1.2	1.3	3.2	0.5	-	-	-	-	-
	520	59.9	57.4	1.0	5.3	8.8	0.9	22.7	43.0	19.3	0.88	0.87
	540	60.2	71.1	0.8	11.0	11.4	1.6	31.7	23.7	20.7	0.85	0.79
**Fe/C**	500	21.2	17.0	1.3	1.0	2.0	0.3	-	-	-	-	-
	520	31.1	37.3	0.8	5.2	5.1	1.0	9.1	44.8	34.8	0.86	0.31
	540	51.7	86.0	0.6	18.5	11.4	2.1	24.1	31.3	12.8	0.88	0.72

**Table 2:** The catalyst porosity analysis by the BET method.

Material	BET Specific Surface Area (m^2^ g^−1^)	Average Pore Volume (cm^3^ g^−1^)	Average Pore Diameter, 4V/A (nm)
Co/C	75.7 ± 0.3	0.225	13.6
Co-Fe/C	79.6 ± 0.3	0.225	13.3
Fe/C	73.3 ± 0.2	0.230	14.3

**Table 3:** Comparing particle size distribution in used catalyst samples by a *t*-test.

Catalyst	Smaller Particles		Larger Particles		*t*-Value
	Mean Size (nm)	s.d. (σ)	Mean Size (nm)	s.d. (σ)	
**Co/C**	6.1	1.7	12.3	4.1	24.0
**Fe/C**	4.2	0.3	9.0	1.1	73.5
**Co/Fe/C**	4.9	1.8	19.2	7.2	−33.5

**Table 4:** Calculated phase composition of used catalyst samples by RQA from XRD analysis.

Analysis	Property	Co/C		Co-Fe/C		Fe/C	
		Phase	(%)	Phase	(%)	Phase	(%)
**XRD** **(spectral data)**	Metallic species	Co (FCC) * Co (HCP) **	62.3 37.7	Co (FCC) α-Fe (FCC) *	12.5 33.1	α-Fe (FCC) *	29.1
	Nano-alloys	–	–	Fe_3_Co	14.0	–	–
	Carbides	–	–	Fe_3_C^#^	30.9	Fe_3_C	48.1
	Oxides	–	–	Fe_3_O_4_*^θ^*	9.5	Fe_3_O_4_	22.8
**RQA** **(statistical data)**	R_(expected)_, (R_exp_)	5.6		5.0		6.5	
	R_(profile)_, (R_p_)	5.8		4.1		7.3	
	R_(weighted profile)_, (R_wp_)	7.4		5.3		9.4	
	GOF*^β^*	1.8		1.1		2.1	

FCC = Face-centered cubic crystal structure (*predominant phase); HCP = hexagonal close packed crystal structure (**minor phase); Fe_3_C^#^ = Cohenite; Fe_3_O_4_*^θ^* = Magnetite; GOF*^β^* = goodness of fit (χ^2^) = (R_wp_/R_exp_)^2^ ≈ 1; R-factors: the Rietveld algorithm optimizes the model function to minimize the weighted sum of squared differences between the observed and computed intensity values; R_p_ = minimized quantity during fitting procedures (by least-squares); R_exp_ = expected R, or the ‘‘best possible “R_wp_” factor; R_wp_ = weighted profile (R-factor); weighted to emphasize peak intensity over background.

**Table 5:** The calculated catalysts’ H_2_ utilization efficiency at various temperatures.

Catalyst	T (K)	H_2_O		Selectivity (mol. %)		H_2_ Efficiency (%)		Ratio
		(cm^3^)	moles	C_5+_	CH_4_	C_5+_	CH_4_	(C_5+_):CH_4_
**Co/C**	500	14.0	0.8	97.8	1.9	15.6	0.5	34.3
	520	41.0	2.3	91.2	5.9	43.7	4.2	10.3
	540	38.0	2.1	68.7	16.4	34.5	12.3	2.8
**Co-Fe/C**	520	33.0	1.8	85.0	8.8	33.8	5.2	6.4
	540	27.0	1.5	76.1	11.4	26.3	5.9	4.5
**Fe/C**	520	21.0	1.2	88.7	5.1	22.4	1.9	11.6
	540	15.0	0.8	68.2	11.4	14.3	3.6	4.0

**Table 6:** The summary properties of the fresh catalysts (averaged values).

Catalyst	Mean C Amount (%)		Metal Particle Size (nm)	BET Surface Area (m^2^ g^−1^)
	G-Phase	D-Phase		
**Co/C**	65	35	11	76
**50%Co-50%Fe/C**	64	36	14	80
**Fe/C**	65	35	11	73

## Supplementary Materials

Supplementary File 1

## Abbreviations

The following abbreviations have been used in this manuscript:

ASF	Anderson–Schulz–Flory distribution
BET	Brunauer-Emmett-Teller method for specific surface area analysis
BJH	Barrett-Joyner-Halenda (porosity analysis model)
CNFs	Carbon nanofilaments
CNTs	Carbon nanotubes
CVD	Carbon-vapor deposition
D	Disordered or amorphous carbon
DBD	Dielectric-barrier discharge (plasma)
EDX	Energy dispersive X-ray spectroscopy
FCC	Face centred cubic crystal structure
FTS	Fischer–Tropsch synthesis
G	Graphitic carbon
G(2H)	Graphite of the hexagonal crystal structure
GC	Gas chromatography
GHSV	Gas hourly space velocity
HCP	Hexagonal closed packing crystal structure
LT-FTS	Low-temperature Fischer–Tropsch synthesis
NTP	Non-thermal plasma reactor
PEBA	Pulsed electron beam ablation
PGD	Plasma-glow discharge
r	Measured feedstock gases consumed in FTS reaction (% by %) as a ratio (H_2_:CO)
RQA	Rietveld quantitative analysis
SLPM	Standard litres per minute
STP	Standard temperature and pressure
SEM	Scanning electron microscopy
SPS	Suspension plasma-spray technology
TEM	Transmission electron microscopy
TOS	Time-on-stream
WGS	Water-gas shift
XPS	X-ray photoelectron spectroscopy
XRD	X-ray diffraction analysis
3-φ-CSTSR	Three-phase continuously-stirred-tank slurry reactor
