# Supplementary material for: Use of Plasma-Synthesized Nano-Catalysts for CO Hydrogenation in Low-Temperature Fischer–Tropsch Synthesis: Effect of Catalyst Pre-Treatment
Source: Nanomaterials (Basel). 2018 Oct 12;8(10):822. doi: 10.3390/nano8100822 (PMC6215254; doi:10.3390/nano8100822)
Supplement: Supplementary File 1 [file nanomaterials-08-00822-s001.pdf]

Highlights

- Fe, Co and Fe-Co thermal plasma-synthesized formulations as low-T FTS catalysts
- Optimization of the pretreatment protocol of said FTS catalytic formulations
- For Fe catalysts the simultaneous presence of oxides & carbides increases activity
- Catalysts exhibit nearly constant BET specific surface and no sintering over TOS
- H_2/CO uptake proved to be decreasing over temperature increase
